# A new approach to silicon rhodamines by Suzuki–Miyaura coupling – scope and limitations

**DOI:** 10.3762/bjoc.15.250

**Published:** 2019-10-29

**Authors:** Thines Kanagasundaram, Antje Timmermann, Carsten S Kramer, Klaus Kopka

**Affiliations:** 1 Division of Radiopharmaceutical Chemistry, German Cancer Research Center (DKFZ), Im Neuenheimer Feld 280, 69120 Heidelberg, Germany; 2 Institute of Inorganic Chemistry, Im Neuenheimer Feld 270, 69120 Heidelberg, Germany; 3 German Cancer Consortium (DKTK), Heidelberg, Germany

**Keywords:** cross coupling, fluorescent dyes, near-infrared (NIR) dyes, silicon rhodamines, Suzuki–Miyaura coupling

## Abstract

**Background:** Silicon rhodamines are of particular interest because of their advantageous dye properties (fluorescence- and biostability, quantum efficiency, tolerance to photobleaching). Therefore, silicon rhodamines find frequent application in STED (stimulated emission depletion) microscopy, as sensor molecules for, e.g., ions and as fluorophores for the optical imaging of tumors. Different strategies were already employed for their synthesis. Because of just three known literature examples in which Suzuki–Miyaura cross couplings gave access to silicon rhodamines in poor to moderate yields, we wanted to improve these first valuable experimental results.

**Results:** The preparation of the xanthene triflate was enhanced and several boron sources were screened to find the optimal coupling partner. After optimization of the palladium catalyst, different substituted boroxines were assessed to explore the scope of the Pd-catalyzed cross-coupling reaction.

**Conclusions:** A number of silicon rhodamines were synthesized under the optimized conditions in up to 91% yield without the necessity of HPLC purification. Moreover, silicon rhodamines functionalized with free acid moieties are directly accessible in contrast to previously described methods.

## Introduction

Silicon rhodamines are versatile fluorescent dyes that found extensive use in super-resolution microscopy [1–8] and as probes for targeting various biomolecules [9–12] or sensors for metal ions [13–17], pH [15], voltage [18] or metabolites [19–22]. Since our group is interested in synthesizing new tumor tracers for intraoperative imaging of cancerous lesions, we were interested in silicon rhodamines due to their fluorescence properties in the biological window (650 nm to 1350 nm). While clinically approved fluorescence dyes like ICG (indocyanine green, *M*_w_ = 775 g/mol) have a high molecular weight and could therefore alter pharmacokinetic or -dynamic properties of the tumor tracers, silicon rhodamines are relatively small and already examined as fluorophores for the optical imaging of tumors. Using silicon rhodamine SiR700 a more enhanced tumor-to-background ratio in optical imaging could be achieved compared to the cyanine based dyes Cy5.5 and Alexa Fluor^®^ 680 [23]. Moreover, silicon rhodamines demonstrated in in vivo imaging experiments excellent fluorescence properties and biostabilities [23] as well as exhibited high quantum efficiencies with high tolerance to photobleaching [24]. A silicon rhodamine antibody conjugate could also be successfully applied for optical imaging of a xenograft tumor (human malignant meningioma) in a mouse model [24]. Again, in direct comparison with the cyanine dye Cy5.5, the silicon rhodamine conjugate showed no fading indicating that silicon rhodamine dyes are more suitable for long time observation than cyanine-based fluorophores [24].

**Scheme 1:**
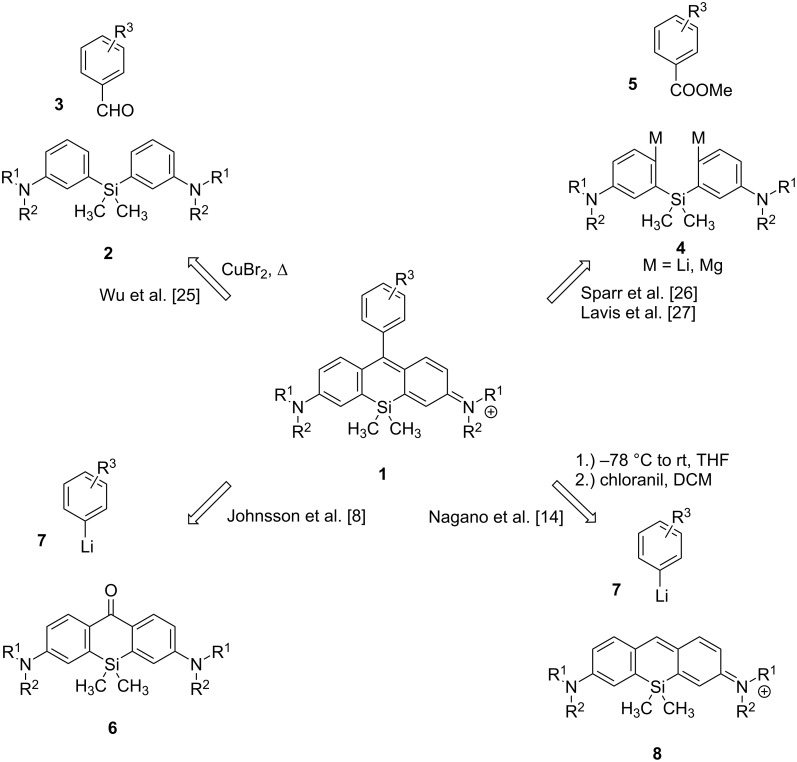
Different synthetic approaches to silicon rhodamine dyes.

Different synthetic approaches were established to form the silicon rhodamine framework **1** (Scheme 1). While the group of Wu used a copper(II) bromide-catalyzed solvent-free condensation of a diarylsilane **2** with various benzaldehydes **3** [25], Sparr and Fischer added the double Grignard reagent **4** to methyl esters **5** [26]. A similar approach was established by Lavis, herein electrophiles (anhydrides or esters) were added to lithium or magnesium organyls **4** [27]. Johnsson and co-workers could establish dye formation by addition of aryllithium **7** to the silicon xanthone **6** [8]. A related strategy, adding lithium compound **7** to a preformed tricyclic system **8**, was used by Nagano et al. to synthesize the Ge and Sn rhodamine analogues [14].

**Scheme 2:**
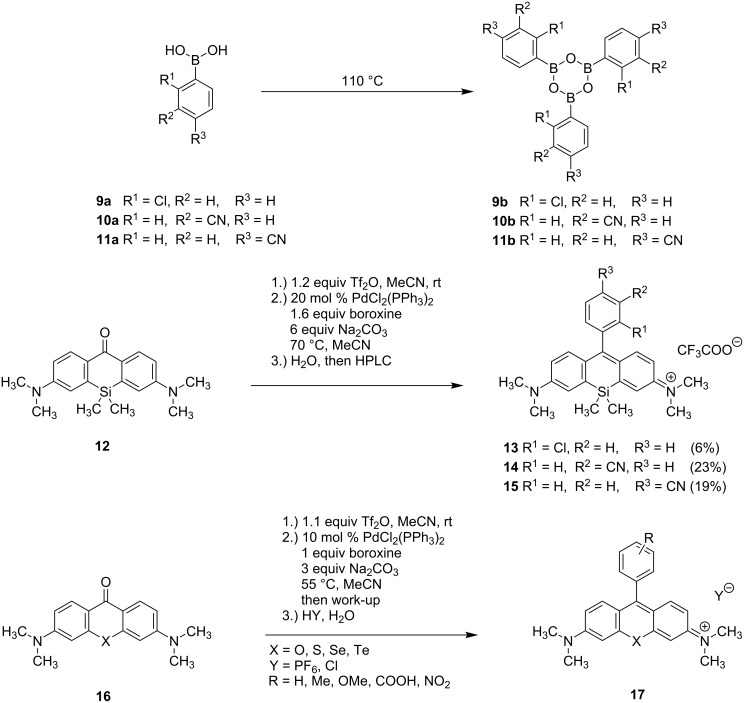
Previous work from Calitree [29] and Urano [22,28] on the Suzuki–Miyaura coupling of triflates, derived from xanthones **12** and **16**, with boroxines.

In a recent publication, Urano et al. synthesized the rhodamines **13**–**15** by coupling the triflate of xanthone **12** with boroxines **9b**–**11b** (Scheme 2) [22,28]. Hereby, the boroxines **9b**–**11b** were accessible by thermal dehydration of the corresponding boronic acids **9a**–**11a**. With this procedure product **13** was obtained in only 6% yield, which is presumably due to a competing coupling reaction of the boroxine moiety of **9b** with the chlorine atom of **9b** or sterical reasons (the chlorine in 2’-position might lead to repulsion during the cross-coupling reaction). The reaction of the triflate with cyano-substituted phenylboroxines **10b** and **11b** led to silicon rhodamine dyes **14** and **15** in poor yields of 23 and 19%, respectively. The reaction conditions applied for the cross coupling of the triflate were similar to those published by Calitree and Detty for the coupling of the triflates derived from the O, S, Se, and Te-xanthones **16** with various phenylboroxines (bearing nitro, carboxylic acid, methyl and methoxy substituents) [29]. Here yields of 53–79% were obtained (for O and S analogues; 85–99% yields based on recovered starting material (brsm)). Since the yields reported by Urano for the Si-analogous Suzuki reactions were much lower (6–23%) [22], we wanted to examine if the aforementioned substrates were outliers and a cross-coupling reaction could be a valuable approach to silicon rhodamines. Thus, we aimed at the optimization of coupling conditions as well as evaluation of the best boron compounds for coupling. Since carboxylic acid-substituted dyes like compound **17** (X = Si, R = COOH) can be easily coupled to tumor binding vectors, we wanted to investigate if these dyes are also accessible by Suzuki–Miyaura coupling.

## Results and Discussion

### Optimization of reaction conditions

**Scheme 3:**
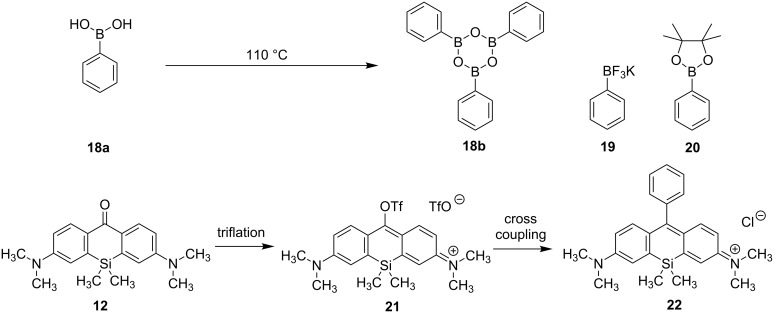
Optimization of cross-coupling conditions of triflate **21**, derived from Si-xanthone **12**, with boron species **18b**, **19** and **20** (see Table 1).

At first we investigated the effects of different catalysts and boron compounds on the synthesis of silicon rhodamine **22** via Suzuki–Miyaura cross coupling (Scheme 3, Table 1). Triflate **21** was obtained without further purification from **12** by addition of triflic anhydride in dry acetonitrile. Boroxine **18b** was formed by heating of boronic acid (**18a**) at 110 °C because it was shown by Calitree and Detty that free boronic acid leads to the destruction of the triflate, resulting in the corresponding xanthone [29]. Applying standard conditions on xanthone **12** by treatment with triflic anhydride in dry acetonitrile and subsequent addition of base, catalyst and boroxine **18b** yielded the desired fluorophore **22** in 41% yield together with unreacted xanthone **12** (Table 1, entry 1). Since the initial triflate formation to **21** was unreliable and often incomplete, leading to lower yields, Comins reagent was investigated as an alternative triflation reagent. Notably, the use of Comins reagent showed no transformation from the yellow xanthone **12** to the deep blue triflate **21** at all (Table 1, entry 2). Exchange of anhydrous acetonitrile by anhydrous dichloromethane, which was removed in vacuo prior to coupling, provided triflate **21** as a blue salt without xanthone residues, hereby the yield could be slightly enhanced but still the conditions of the coupling reaction led to some back reaction of **21** to **12** (Table 1, entry 3). While the use of PdCl_2_(PPh_3_)_2_ was successful in the synthesis of chalcogenorhodamine dyes [29], the usage of that catalyst gave just low yields when applied in the synthesis of the silicon analogues (Scheme 2) [22]. Although Pd(PPh_3_)_4_ was not found to be an effective catalyst for the synthesis of rhodamine and rosamine dyes as well as for their selenium or tellurium analogous [29], the usage of that Pd(0) catalyst showed yields comparable with those obtained with PdCl_2_(PPh_3_)_2_ (Table 1, entry 4). The exchange of sodium carbonate with cesium carbonate resulted in no reaction at all (Table 1, entry 5). Whereby usage of potassium phenyltrifluoroborate (**19**) resulted in a yield comparable to boroxine **18b** (Table 1, entry 6), usage of pinacol ester **20** showed no reaction in the cross-coupling reaction (Table 1, entry 7). Although described optimizations of the reaction conditions could lead to the silicon rhodamine **22** in moderate yields, an inseparable impurity of the cationic fluorophore was detected. After identifying this impurity as the tetraphenylphosphonium cation, we exchanged the triphenylphosphine ligand of the catalyst with dppf (1,1'-bis(diphenylphosphino)ferrocene). Remarkably, not only the yield was increased with PdCl_2_(dppf) from 49% to 67%, even the dye **22** was obtained with high purity after column chromatography without the necessity of further HPLC purification (Table 1, entry 8).

**Table 1:** Optimization of cross-coupling conditions of triflate **21**, derived from Si-xanthone **12**, with boron species **18b**, **19** and **20**.

Entry	Triflation	Cross coupling			Yield (**22**)
		Catalyst (10 mol %)	Boron species (1 equiv)	Conditions	

1	1.1 equiv Tf_2_O, MeCN, rt, 20 min	PdCl_2_(PPh_3_)_2_	**18b**	3 equiv Na_2_CO_3_, MeCN, 70 °C, overnight	41%^a^
2	1 equiv Comins’ reagent (5-Cl-2-pyridyl-NTf_2_), MeCN, rt, 1 h	–	–	–	–
3	1.1 equiv Tf_2_O, DCM, rt, 20 min, then evaporation	PdCl_2_(PPh_3_)_2_	**18b**	3 equiv Na_2_CO_3_, MeCN, 70 °C, overnight	49%^a^, 80%^a,b^
4	1.1 equiv Tf_2_O, DCM, rt, 20 min, then evaporation	Pd(PPh_3_)_4_	**18b**	3 equiv Na_2_CO_3_, MeCN, 70 °C, overnight	39%^a^, 82%^a,b^
5	1.1 equiv Tf_2_O, DCM, rt, 20 min, then evaporation	PdCl_2_(PPh_3_)_2_	**18b**	3 equiv Cs_2_CO_3_, MeCN, 70 °C, overnight	n.r.
6	1.1 equiv Tf_2_O, DCM, rt, 20 min, then evaporation	PdCl_2_(PPh_3_)_2_	**19**	3 equiv Na_2_CO_3_, MeCN, 70 °C, overnight	48%^a^
7	1.1 equiv Tf_2_O, DCM, rt, 20 min, then evaporation	PdCl_2_(PPh_3_)_2_	**20**	3 equiv Na_2_CO_3_, MeCN, 70 °C, overnight	n.r.
8	1.1 equiv Tf_2_O, DCM, rt, 20 min, then evaporation	PdCl_2_(dppf)	**18b**	3 equiv Na_2_CO_3_, MeCN, 70 °C, overnight	67%, 73%^b^

^a^Corrected yield, contamination with [PPh_4_]^+^. ^b^Based on recovered starting material (brsm) **12**.

### Exploration of substrate scope

**Scheme 4:**
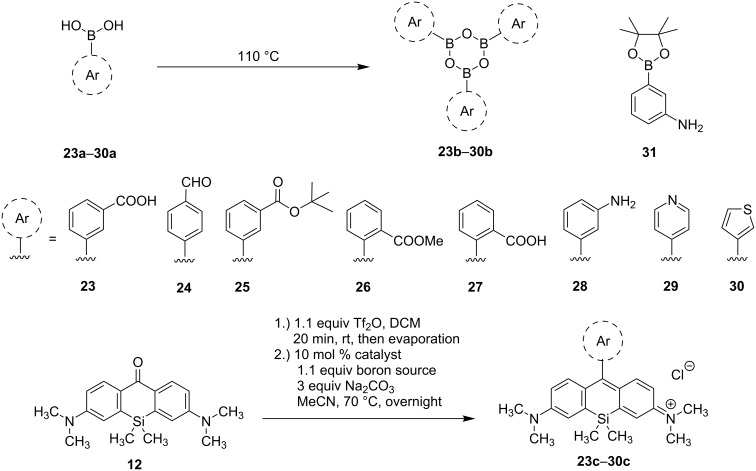
Coupling reactions of silicon xanthone **12** with different boron species (**23b**–**30b**, **31**).

Next we explored the substrate scope of the Suzuki–Miyaura coupling by screening commercially available boronic acids (Scheme 4, Table 2). Hereby, PdCl_2_(dppf) was also tested in order to suppress the formation of the inseparable phosphonium cation species. At first, we investigated the use of 3-boronobenzoic acid (**23a**) that should lead to a rhodamine suitable for coupling to a tumor vector, but boroxine **23b** was converted to **23c** with PdCl_2_(PPh_3_)_2_ in poor yields (Scheme 4 and Table 2, entry 1). However, PdCl_2_(dppf) performed better and led to the acid-substituted silicon rhodamine **23c** in a moderate yield of 31% (56% brsm) (Table 2, entry 2). The moderate yield might be explained with the destruction of the triflate by the acid moiety of **23c**. In order to prevent the destruction of the initially formed triflate **21**, 4-boronobenzaldehyde (**24a**) was intended as a coupling substrate but yielded silicon rhodamine **24c** only in traces (Table 2, entry 3). Usage of the *tert*-butyl-protected boronobenzoic acid **25a**, or its boroxine counterpart **25b**, respectively, gave fluorophore **25c** suitable for later coupling reactions in reasonable yields of 43% and 53%, depending on the catalyst used (Table 2, entries 4 and 5). Again, the reaction catalyzed by PdCl_2_(dppf) resulted in an enhanced yield compared to catalysis with PdCl_2_(PPh_3_)_2_. Next we aimed at the synthesis of a silicon rhodamine bearing an acid function in 2’-position. With a less bulky methyl ester in the 2’-position of the phenylboroxine, the transmetalation and the new bond formation through reductive elimination should be less hindered, but remarkably, no reaction was observed either with the methyl ester **26b** or the free acid **27b** (Table 2, entries 6 and 7). Next we explored if amino-substituted silicon rhodamine **28c** is accessible via Pd-catalysis. The resulting rhodamine **28c** could be a possible substrate for the conversion into an azide and follow-up click reactions with alkyne-substituted tumor vectors. While heating of amine **28a** to the corresponding boroxine **28b** lead to formation of a brown solid (presumably due to degradation), the reaction of triflate **21** with the pinacol ester **31** showed no product formation at all (Table 2, entries 8 and 9). Since we were able to investigate the functional group tolerance of the coupling reaction, we shifted our focus towards heterocyclic boronic acids as substrates. Since 4’-pyridinyl- [27,30] and 3’-thienyl- [27,31–33] substituted silicon rhodamines are already known, we investigated the synthesis of these dyes by Suzuki–Miyaura cross coupling. Firstly, pyridinylboronic acid **29a** was used as a substrate after heating at 110 °C, but no conversion was observed presumably due to the formation of an internal salt (protonated pyridine ring and deprotonated boronic acid) and ensuing difficult formation of boroxine **29b** (Table 2, entry 10). Switching to the neutral heterocyclic boronic acid **30a**, the corresponding thienyl-substituted silicon rhodamine **30c** could be obtained in 37% (56% brsm) yield with the PdCl_2_(PPh_3_)_2_ catalyst. Remarkably, the yield could be clearly enhanced by catalysis with PdCl_2_(dppf) and the thienyl-substituted fluorophore **30c** could subsequently be synthesized in 91% yield.

**Table 2:** Coupling reactions of silicon xanthone **12** with different boron species (**23b**–**30b**, **31**).

Entry	Boron source	Catalyst	Yield

1	**23b**	PdCl_2_(PPh_3_)_2_	5%^a^, 46%^a,b^ (**23c**)
2	**23b**	PdCl_2_(dppf)	31%, 56%^b^ (**23c**)
3	**24b**	PdCl_2_(PPh_3_)_2_	traces (**24c**)
4	**25b**	PdCl_2_(PPh_3_)_2_	43%^a^, 62%^a,b^ (**25c**)
5	**25b**	PdCl_2_(dppf)	53%, 66%^b^ (**25c**)
6	**26b**	PdCl_2_(PPh_3_)_2_	n.r.
7	**27b**	PdCl_2_(PPh_3_)_2_	n.r.
8	**28b**	PdCl_2_(PPh_3_)_2_	n.r.
9	**31**	PdCl_2_(PPh_3_)_2_	n.r.
10	**29b**	PdCl_2_(PPh_3_)_2_	n.r.
11	**30b**	PdCl_2_(PPh_3_)_2_	37%^a^, 56%^a,b^ (**30c**)
12	**30b**	PdCl_2_(dppf)	91% (**30c**)

^a^Corrected yield, contamination with [PPh_3_Ar]^+^. ^b^Based on recovered starting material (brsm) **12**.

Table 3 compares the reaction outcome of the silicon rhodamine synthesis via Suzuki coupling with other employed methods: synthesis of the phenyl-substituted silicon rhodamine **22** by Suzuki cross coupling affords the product in a similar yield compared to the addition of phenyllithium to xanthone **12** or the attack of the double metallated bis-aniline **4** (R^1^ = R^2^ = Me, M = Mg) to the benzoic acid methyl ester [26]. However, the cross coupling of triflate **21** with boroxine **25b** led to the ester-substituted rhodamine **25c** in a reasonable yield of 53% (66% brsm) while the addition of the lithiated *tert*-butyl 3-bromobenzoate gave the fluorophore **25c** in only 7% yield. Finally, the cross-coupling reaction of **12** and **30b** to rhodamine **30c** clearly outperforms the addition of lithiated 2-bromothiophene to xanthone **12** since 2-bromothiophene might also undergo lithiation in 5-position in competition to the halogen metal exchange (in general multiple halogenated aryls are problematic nucleophiles for these addition reactions).

**Table 3:** Comparison of common methods for silicon rhodamine synthesis.

Method →	Addition of lithium organyl to **12**^a^	Suzuki–Miyaura cross coupling	Attack of **4** (R^1^ = R^2^ = Me, M = Mg) to **5** (R^3^ = H)
Fluorophore ↓			

phenyl-substituted SiR (**22**)	72%	67%, 73%^b^	72% [26]
*tert*-butylbenzoic acid-substituted SiR (**25c**)	7%	53%, 66%^b^	–
thienyl-substituted SiR (**30c**)	77%	91%	–

^a^Conditions: 7 equiv aryl bromide, 14 equiv *t*-BuLi, THF, −78 °C, 30 min, then 1 equiv **12** at −78 °C to rt, overnight, then aq HCl, work-up, purification with DCM/MeOH 99:1 to 9:1. ^b^Based on recovered starting material (brsm) **12**.

## Conclusion

Since just three literature examples are known to date in which Suzuki–Miyaura cross-coupling reactions gave access to silicon rhodamines in poor to moderate yields (Scheme 2), we wanted to improve these first valuable experimental results. In general, the amount of re-isolated starting material **12** could be significantly reduced when acetonitrile was exchanged with dichloromethane in the triflation reaction to provide triflate **21** neat and more reliable. Screening of different boron species and catalysts showed that, like in the syntheses of O, S, Se, and Te-rhodamines, boroxines were a suitable source, but also potassium trifluoroborates can be taken into consideration for the reaction design, whereas pinacol esters didn’t show any reactivity. While PdCl_2_(PPh_3_)_2_ was a sufficient catalyst for the cross coupling, application of PdCl_2_(dppf) led to clearly enhanced yields: overall the Suzuki–Miyaura cross-coupling reaction gave access to silicon rhodamines with neutral (hetero)aromatic xanthene substituents (phenyl: 67%, respectively 73% brsm; thienyl: 91%) (even though the term ‘dihydrosilaanthracene’ is correct to name the Si-anthracene moiety, the term ‘Si-xanthene’ is widely used in the literature (see e.g. [30]); also the term Si-xanthone (for derivatives of **12**) is established instead of 9-silaanthracen-10(9*H*)-one). The conditions tolerated also the use of the unprotected acid functionality of the boroxine **23b** (**23c**, 31%, respectively 56% brsm), while application of basic boronic acids failed (**28**, **29**), presumably due to unsuccessful boroxine formation. The main advantage of the cross coupling is the access to acid-functionalized fluorophores like **23c** that can be immediately coupled to a molecule of interest (e.g., tumor binding vectors) whereas previously published methodologies need, e.g., an ester, orthoester or oxazoline protecting group for the acid. But also the *tert*-butyl ester-functionalized boroxine **25** is suitable for the cross coupling. With the current catalytic system, coupling of 2-substituted boroxines (**26**, **27**) remains challenging, but optimizing the catalytic system with ligands suitable for coupling of multisubstituted aryls is under current investigation. In conclusion, several silicon rhodamines could be synthesized under the optimized conditions, without the necessity of HPLC purification, in up to 91% yield whereby the free acids are directly accessible in contrast to the three hitherto described methods.

## Supporting Information

File 1 Experimental procedures and NMR spectra of all synthesized compounds as well as photochromic characterization data (fluorescence spectra, quantum yield) of thienyl-substituted silicon rhodamine **30c**.
