# Supplementary material for: A new approach to silicon rhodamines by Suzuki–Miyaura coupling – scope and limitations
Source: Beilstein J Org Chem. 2019 Oct 29;15:2569–76. doi: 10.3762/bjoc.15.250 (PMC6839552; doi:10.3762/bjoc.15.250)
Supplement: File 1 — Experimental procedures and NMR spectra of all synthesized compounds as well as photochromic characterization data (fluorescence spectra, quantum yield) of thienyl-substituted silicon rhodamine 30c. [file Beilstein_J_Org_Chem-15-2569-s001.pdf]

BEILSTEIN JOURNAL OF ORGANIC CHEMISTRY

Supporting Information

for

A new approach to silicon rhodamines by Suzuki–Miyaura coupling – scope and limitations

Thines Kanagasundaram, Antje Timmermann, Carsten S. Kramer and Klaus Kopka

Beilstein J. Org. Chem. **2019,** *15,* 2569–2576. doi:10.3762/bjoc.15.250

Experimental procedures and NMR spectra of all synthesized compounds as well as photochromic characterization data (fluorescence spectra, quantum yield) of thienyl-substituted silicon rhodamine 30c

License and Terms: This is a supporting information file under the terms of the Creative Commons Attribution License (http://creativecommons.org/licenses/by/4.0). Please note that the reuse, redistribution and reproduction in particular requires that the authors and source are credited.
The license is subject to the *Beilstein Journal of Organic Chemistry* terms and conditions: (https://www.beilstein-journals.org/bjoc)

Table of contents

1. General remarks.. S2

2. Synthesis.. S4

2.1 Boron sources for the Suzuki–Miyaura cross-coupling reactions.. S4

2.2 General procedure: synthesis of boroxines.. S4

2.3 Synthesis of 4,4‘-methylenebis(3-bromo-*N*,*N*-dimethylaniline).. S5

2.4 Synthesis of xanthone **12**.. S5

2.5 Suzuki–Miyaura cross coupling reactions – general procedure.. S7

2.5.1 Synthesis of Si-rhodamine **22**.. S7

2.5.2 Synthesis of Si-rhodamine **22**.. S8

2.5.3 Synthesis of Si-rhodamine **23c**.. S10

2.5.4 Synthesis of Si-rhodamine **25c**.. S11

2.5.5 Synthesis of Si-rhodamine **30c**.. S12

3. NMR-Spectra.. S14

4. UV–vis/NIR-spectrum of compound **30c**.. S19

1. General remarks

Unless otherwise stated reactions requiring exclusion of oxygen and moisture were carried out in heat-gun flasks dried under argon gas atmosphere using the Schlenk-technique.
All **chemicals** and **solvents** were taken from Sigma-Aldrich Laborchemikalien GmbH, abcr GmbH, Acros Organics and were used as received without further purification. Deuterated solvents were used from Deutero GmbH. The catalyst [1,1′-bis(diphenylphosphino)ferrocene]dichloropalladium(II) was obtained from Sigma-Aldrich Laborchemikalien GmbH as a complex with dichloromethane.

NMR spectra were recorded at room temperature on the following spectrometers: 400 MHz and 600 MHz: Bruker Avance III. Chemical shifts are reported in *δ* units relative to chloroform-*d* (δ_H = 7.26; δ_C = 77.2) or methanol-d_4 (δ_H = 3.31; δ_C = 49.0).[1] Analyses followed first order and the following abbreviations were used throughout: s = singlet, d = doublet, t = triplet, dd = doublet of doublet etc., m = multiplet. Coupling constants (*J*) are given in Hz and refer to H,H-couplings.

Mass spectra (MS) were determined in the Institute for Organic Chemistry of the University of Heidelberg under the direction of Dr. Jürgen Gross. The ionization method ESI was applied using spectrometer BrukerApexQe hybrid 9.4 T FT-ICR. High-resolution mass spectra (HRMS) were recorded with a JEOL JMS-700 spectrometer. The molecule ions are reported as mass to charge (*m/z*) relation.

UV–vis/NIR absorption spectrum of **30c** was measured at the Max Planck Institute for Biomedical Research Heidelberg with a Cary 5000 UV–vis–NIR spectrometer by M. Sc. Jessica Matthias (Group of Prof. Stefan Hell). The emission spectrum of **30c** was recorded with a Cary Eclipse Fluorescence spectrometer. All measurements were performed in acetonitrile. The quantum yield was determined with nile blue as a calibration standard.

Analytical thin-layer chromatography (TLC) was carried out on polygram-TLC-plates produced by Machery-Nagel (40 × 80 mm, SIL G/UV254, 0.2 mm layer thickness). Detection was carried out using UV-light (254 nm or 366 nm).

[1] G. R. Fulmer, A. J. M. Miller, N. H. Sherden, H. E. Gottlieb, A. Nudelman, B. M. Stoltz, J. E. Bercaw, K. I. Goldberg, *Organometallics* **2010**, *29*, 2176–2179.

Flash column chromatography was carried out on silica gel (0.032 mm–0.062 mm, produced by Macherey-Nagel) using manual techniques. As mobile phase mixtures of solvents (hexanes/ethyl acetate, dichloromethane/methanol and chloroform/methanol) were used.

Chemical formulas were drawn with ChemDraw Professional 15.0.

2. Synthesis

2.1 Boron sources for the Suzuki–Miyaura cross coupling reactions

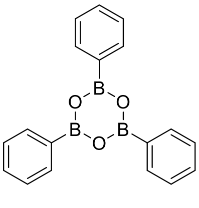

18b

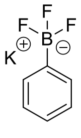

19

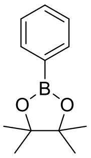

20

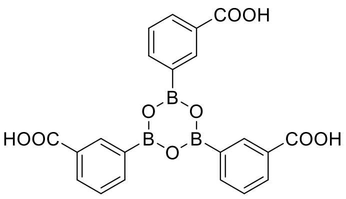

23b

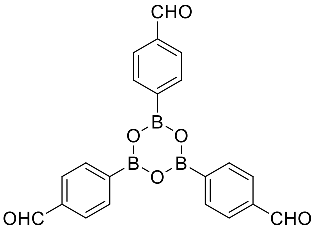

24b

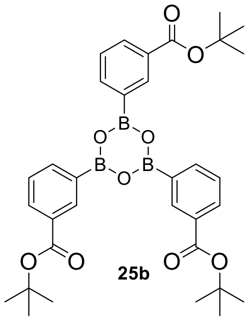

25b

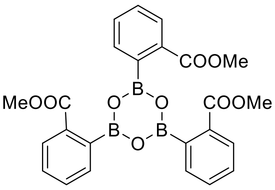

26b

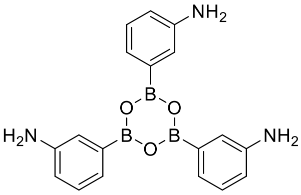

28b

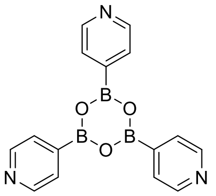

29b

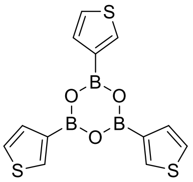

30b

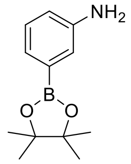

31

2.2 General procedure: synthesis of boroxines[2]

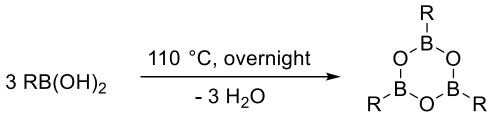

32 **33**

The arylboronic acid (100 mg) was given into a resealable glass vial and was heated up to 110 °C at high vacuum overnight. The obtained boroxine was added to the upcoming Suzuki–Miyaura cross-coupling reactions without further purification and characterization.

[2] B. Calitree, M. Detty, *Synlett* **2010**, 89–92.

2.3 Synthesis of 4,4'-methylenebis(3-bromo-*N*,*N*-dimethylaniline)

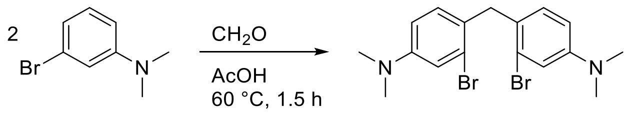

3-Bromo-*N*,*N*-dimethylaniline (**34**) (10.0 g, 50.0 mmol, 1.0 equiv) was dissolved in concentrated acetic acid (80 mL). Formaldehyde (37 wt % in water, with methanol as stabilizer, 10.0 mL, 134 mmol, 2.7 equiv) was added to the colorless solution. The reaction mixture was heated to 60 °C and stirred for 1.5 hours. After complete conversion (monitored by TLC) the solution was allowed to cool down. The excess of acetic acid was evaporated under reduced pressure. A saturated solution of sodium bicarbonate (20 mL) was added carefully to the reaction mixture. The suspension was extracted with ethyl acetate (4 × 50 mL) then the combined organic phases were dried with sodium sulfate and filtered off. The solvent was removed by evaporation in vacuum. The crude product was purified by flash column chromatography (silica gel, *n*-hexane/ethyl acetate 95:5) to afford pale 4,4'-methylenebis(3-bromo-*N*,*N*-dimethylaniline) (**35**) (6.81 g, 16.5 mmol, 66%) as a colorless solid.

^{1}H NMR (400 MHz, $CDCl_3$, 300 K):
δ (ppm) = 6.97 (s, 2H, H_{arom}), 6.86 (d, J = 8.5, 2H, H_{arom}), 6.62 (d, J = 6.8, 2H, H_{arom}), 4.01 (s, 2H, CH_2), 2.92 (s, 12H, CH_3).

R_f value (*n*-hexane/ethyl acetate: 95:5) = 0.30

**$C_{17}H_{20}{}^{79}Br^{81}BrN_2$**	Calculated m/z	found m/z
$[M+H]^+$	413.0046	413.0050

The analytical data are in accordance with the data in the literature.[3]

2.4 Synthesis of xanthone 12

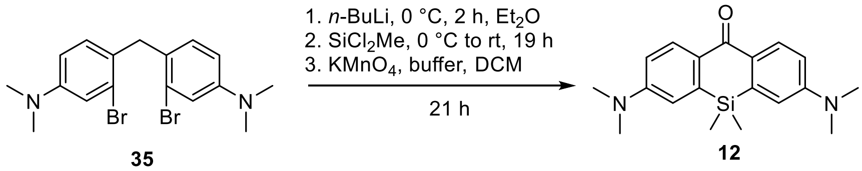

[3] G. Lukinavičius, K. Umezawa, N. Olivier, A. Honigmann, G. Yang, T. Plass, V. Mueller, L. Reymond, I. R. Corrêa, Z.-G. Luo et al., *Nat. Chem.* **2013**, *5*, 132–139.

Under argon atmosphere **35** (1.50 g, 3.64 mmol, 1.0 equiv) was dissolved in dry diethyl ether (80 mL) in a heat-dried round-bottom flask. The colorless solution was cooled down to 0 °C and *n*-BuLi (4.0 mL, 2.5 M in *n*-hexane, 10.2 mmol, 2.8 equiv) was added dropwise. The bright yellow solution was stirred for two hours at 0 °C. Subsequently dichlorodimethylsilane (470 mg, 3.64 mmol, 1.0 equiv) was added dropwise via a syringe to the reaction mixture at 0 °C. A colorless precipitation was observed within few minutes. The solution was allowed to warm up to room temperature and was stirred for further 19 h. After complete conversion (monitored by TLC) the pale yellow mixture was quenched with water and extracted three times with diethyl ether (400 mL). The combined organic phases were washed with brine and were dried with sodium sulfate. After filtration the solvent was removed by evaporating. The subsequent benzylic oxidation was conducted using a procedure published by Bertozzi et al.:[4] The residual brown solid was dissolved in DCM (40 mL) and cooled down to 0 °C. Then a mixture of potassium permanganate (1.73 g, 10.9 mmol, 3.0 equiv) in water (30 mL), a solution of 1 M potassium hydroxide (7.5 mL) and tetrabutylammonium bisulfate (510 mg, 1.50 mmol, 0.4 equiv) was freshly prepared in a separate flask. The dark violet solution was added carefully to the blue DCM solution at 0 °C. The reaction mixture was stirred for 30 minutes at room temperature. The reaction was quenched by addition of acetic acid (7.5 mL). Then sodium sulfite (2.85 g, 22.7 mmol, 6.0 equiv) was added in one portion. The color of the solution changed from brown to dark green immediately. The reaction mixture was diluted with water and the organic phase was separated. The aqueous phase was extracted with ethyl acetate (200 mL) two times and three times with DCM (250 mL) afterwards. The combined organic phases were washed with brine and dried over sodium sulfate. After filtration the solvent was removed under vacuum. The crude product was purified by flash column chromatography (silica gel, *n*-hexane/ethyl acetate 95:5 to 60:40) to afford **12** (614 mg, 1.89 mmol, 52%) as a yellow solid.

^{1}H NMR (400 MHz, $CDCl_3$, 300 K):
δ (ppm) = 8.40 (d, J = 9.0 Hz, 2 H, H_{arom}), 6.85 (d, J = 2.1 Hz, 2 H, H_{arom}), 6.80 (dd, J = 8.7 Hz, 2.6 Hz, 2 H, H_{arom}), 3.10 (s, 12 H, N-CH_3), 0.47 (s, 6 H, Si-CH_3).

^{13}C NMR (400 MHz, $CDCl_3$, 300 K):
δ (ppm) = 185.4, 151.5, 140.6, 131.8, 129.9, 114.4, 113.3, 40.2, -0.9.

R_f value (*n*-hexane/ethyl acetate: 8:2) = 0.18

---

[4] C. Bertozzi, P. Shieh, (2015). Alkyne-activated fluorogenic azide compounds and methods of use thereof. US9410958B2.

$C_{19}H_{24}N_2OSi$	Calculated m/z	found m/z
$[2M+Na]^+$	671.3208	671.3218

The analytical data are in accordance with the data in the literature.[5]

2.5 Suzuki–Miyaura cross coupling reactions – general procedure

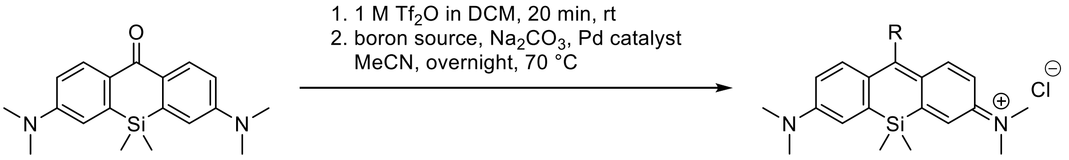

12 **22–30c**

Under argon atmosphere xanthone **12** (1.0 eq.) was dissolved in a heat-dried flask in dry dichloromethane. Trifluoromethanesulfonic anhydride (1.0 equiv, 1 M in DCM) was added to the solution under vigorous stirring. Immediately an intense color change from yellow to dark blue was observed. After 20 minutes of stirring at room temperature dichloromethane was removed under reduced pressure. The boron source (1.1 equiv), the Pd catalyst (0.1 equiv), and sodium carbonate (3.0 equiv) were added to the blue residue. The solids were suspended in anhydrous acetonitrile. The dark blue solution was warmed up to 70 °C and was stirred overnight. After the reaction time the solution was allowed to cool to room temperature. The solvent was completely removed under reduced pressure and was dissolved in dichloromethane. The blue solution was filtrated over a short pad of Celite®. Deionized water was added to the filtrate and the solution was transferred into a separation funnel. The organic phase was separated. The aqueous phase was extracted three times with DCM. The combined organic phases were washed with brine and dried over sodium sulfate. After filtration the solvent was removed under vacuum. The crude product was purified by flash column chromatography to afford the Si-rhodamines as intense blue solids.

2.5.1 Synthesis of Si-rhodamine 22

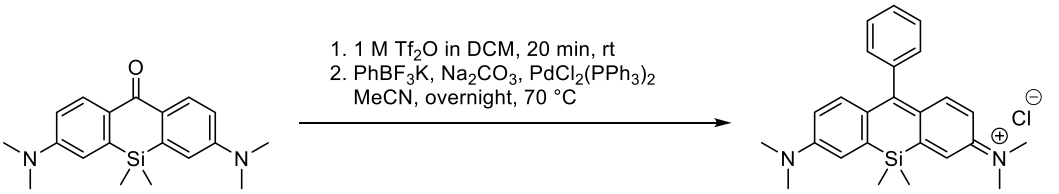

12 **22**

[5] J. L. Bachman, P. R. Escamilla, A. J. Boley, C. I. Pavlich, E. V. Anslyn, *Org. Lett.* **2019**, *21*, 206–209.

The synthesis was conducted as described in the general procedure 2.5. The xanthone **12** (50 mg, 154 µmol, 1.0 equiv) was treated with trifluoromethanesulfonic anhydride (170 µL, 170 µmol, 1 M in DCM, 1.1 equiv) in DCM (1 mL). Potassium phenyltrifluoroborate (28 mg, 154 µmol, 1.0 equiv) was used as boron source and bis(triphenylphosphine)palladium dichloride (11 mg, 15.4 µmol, 0.1 equiv), sodium carbonate (50 mg, 462 µmol, 3.0 equiv) were added and suspended in anhydrous acetonitrile (2 mL). Si-rhodamine **22** (31 mg, 74 µmol, 48%) was obtained as a blue solid after purification by column chromatography (silica gel, dichloromethane/methanol 99:1 to 90:10).

^{1}H NMR (600 MHz, CD_3OD, 300 K):
δ (ppm) = 7.61–7.58 (m, 3 H, H_{arom}), 7.36 (d, *J* = 2.7 Hz, 2 H, H_{arom}), 7.30–7.27 (m, 2 H, H_{arom}), 7.16 (d, *J* = 9.6 Hz, 2 H, H_{arom}), 6.77 (dd, *J* = 9.6 Hz, J = 2.8 Hz, 2 H, H_{arom}), 3.34 (s, 12 H, N-CH_3), 0.60 (s, 6 H, Si-CH_3).

^{13}C NMR (151 MHz, CD_3OD, 300 K):
δ (ppm) = 171.0, 155.6, 149.6, 143.2, 140.6, 130.4, 129.8, 129.3, 129.0, 122.2, 114.9, 40.9, −1.1.

R_f value (dichloromethane/methanol: 9:1) = 0.53

$C_{25}H_{29}N_2Si^+$	Calculated m/z	found m/z
$[M]^+$	385.2095	385.2100

The analytical data are consistent with the data in the literature.[6]

2.5.2 Synthesis of Si-rhodamine 22

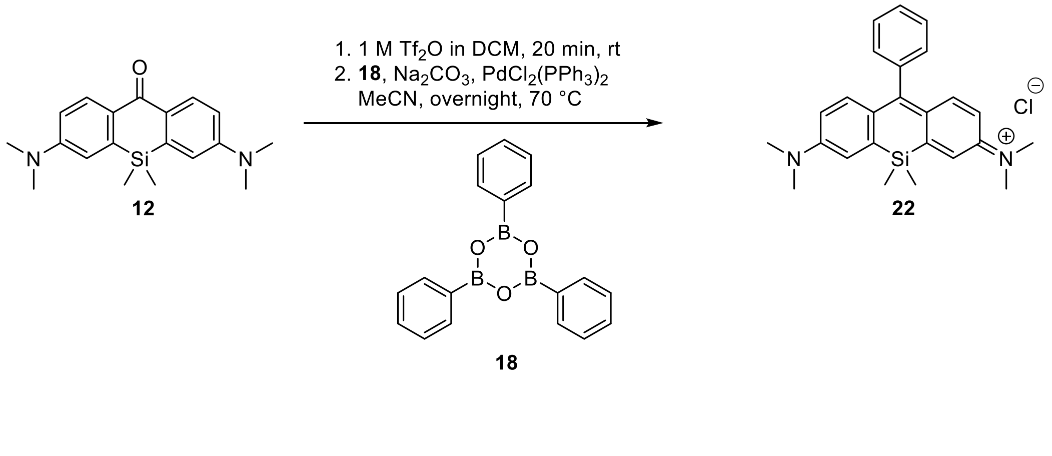

[6] C. Fischer, C. Sparr, *Angew. Chem. Int. Ed.* **2018**, *57*, 2436–2440.

The synthesis was conducted as described in the general procedure 2.5. Xanthone **12** (50 mg, 154 µmol, 1.0 equiv) was treated with trifluoromethanesulfonic anhydride (170 µL, 170 µmol, 1 M in DCM, 1.1 equiv) in DCM (1 mL). Phenylboroxine **18** (48.1 mg, 154 µmol, 1.0 equiv) was used as boron source and bis(triphenylphosphine)palladium dichloride (11 mg, 15.4 µmol, 0.1 equiv), sodium carbonate (49 mg, 462 µmol, 3.0 equiv) were added and suspended in anhydrous acetonitrile (2 mL). Si-rhodamine **22** was obtained as a blue solid in 49%[a] yield (52 mg, 123 µmol, 80%[a] brsm) after purification via column chromatography (silica gel, dichloromethane/methanol 99:1 to 90:10).

Using the same procedure 2.5 and tetrakis(triphenylphosphine)palladium(0) (17.7 mg, 15.4 µmol, 0.1 equiv) as a catalyst provided rhodamine **22** in 39%[a] yield (53.2 mg, 126 µmol, 82% brsm).

Using the same procedure 2.5 and [1,1′-bis(diphenylphosphino)ferrocene]dichloropalladium(II) (13.0 mg, 15.9 µmol, 0.1 equiv) as a catalyst provided rhodamine **22** in 67% yield (47.3 mg, 112 µmol, 73% brsm).

[a]corrected yield, impurification with $[PPh_4]^+$

^{1}H NMR (600 MHz, CD_3OD, 300 K):
δ (ppm) = 7.61–7.58 (m, 3 H, H_{arom}), 7.36 (d, *J* = 2.7 Hz, 2 H, H_{arom}), 7.30–7.27 (m, 2 H, H_{arom}), 7.16 (d, *J* = 9.6 Hz, 2 H, H_{arom}), 6.77 (dd, *J* = 9.6 Hz, J = 2.8 Hz, 2 H, H_{arom}), 3.34 (s, 12 H, N-CH_3), 0.60 (s, 6 H, Si-CH_3).

^{13}C NMR (151 MHz, CD_3OD, 300 K):
δ (ppm) = 171.0, 155.6, 149.6, 143.2, 140.6, 130.4, 129.8, 129.3, 129.0, 122.2, 114.9, 40.9, −1.1.

R_f value (dichloromethane/methanol: 9:1) = 0.53

**$C_{25}H_{29}N_2Si^+$**	Calculated m/z	found m/z
$[M]^+$	385.2095	385.2100

The analytical data are consistent with the data in the literature.[6]

2.5.3 Synthesis of Si-rhodamine 23c

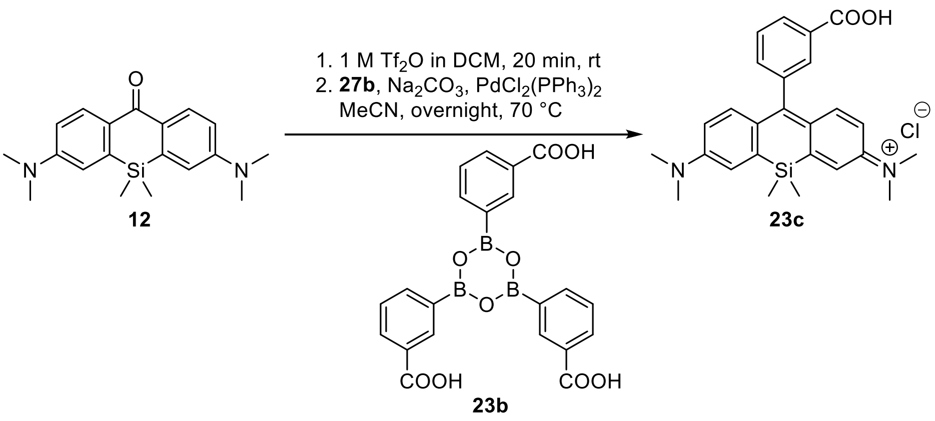

The synthesis was conducted as described in the general procedure 2.5. Xanthone **12** (50 mg, 154 µmol, 1.0 equiv) was treated with trifluoromethanesulfonic anhydride (170 µL, 170 µmol, 1 M in DCM, 1.1 equiv) in DCM (1 mL). Boroxine **23b** (73.0 mg, 154 µmol, 1.0 equiv) was used as boron source and bis(triphenylphosphine)palladium dichloride (11 mg, 15.9 µmol, 0.1 eq.), sodium carbonate (49 mg, 462 µmol, 3.0 equiv) were added and suspended in anhydrous acetonitrile (2 mL). Si-rhodamine **23c** was obtained as a blue solid in 5%[a] yield (22.4 mg, 48.2 µmol, 46%[a] brsm) after purification via column chromatography (silica gel, dichloromethane/methanol 99:1 to 80:20).

Using the same procedure 2.5 and [1,1′-bis(diphenylphosphino)ferrocene]dichloropalladium(II) (13.0 mg, 15.9 µmol, 0.1 equiv) as a catalyst provided rhodamine **23c** in 31% yield (40.1 mg, 86.2 µmol, 56% brsm) after purification by column chromatography (silica gel, chloroform/methanol 99:1 to 80:20).

[a]corrected yield, impurification with $[PPh_3(Ph\text{-}COOH)]^+$

^{1}H NMR (600 MHz, CD_3OD, 300 K):
δ (ppm) = 8.22 (d, *J* = 7.2 Hz, 1 H, H_{arom}), 7.88 (s, 1 H, H_{arom}), 7.70 (d, *J* = 7.7 Hz, 1 H, H_{arom}), 7.52 (d, *J* = 7.5 Hz, 1 H, H_{arom}), 7.38 (d, *J* = 2.8 Hz, 2 H, H_{arom}), 7.08 (d, *J* = 9.7 Hz, 2 H, H_{arom}), 6.79 (d, *J* = 8.7 Hz, 2 H, H_{arom}), 3.35 (s, 12 H, N-CH_3), 0.61 (s, 6 H, Si-CH_3).

^{13}C NMR (151 MHz, CD_3OD, 300 K):
δ (ppm) = 185.4, 168.9, 155.7, 149.6, 142.9, 140.9, 134.7, 132.2, 131.3, 130.9, 129.7, 128.8, 122.4, 115.1, 40.9, −1.1.

R_f value (dichloromethane/methanol: 9:1) = 0.13

$C_{26}H_{29}N_2O_2Si^+$	Calculated m/z	found m/z
$[M]^+$	429.1993	429.1996

2.5.4 Synthesis of Si-rhodamine 25c

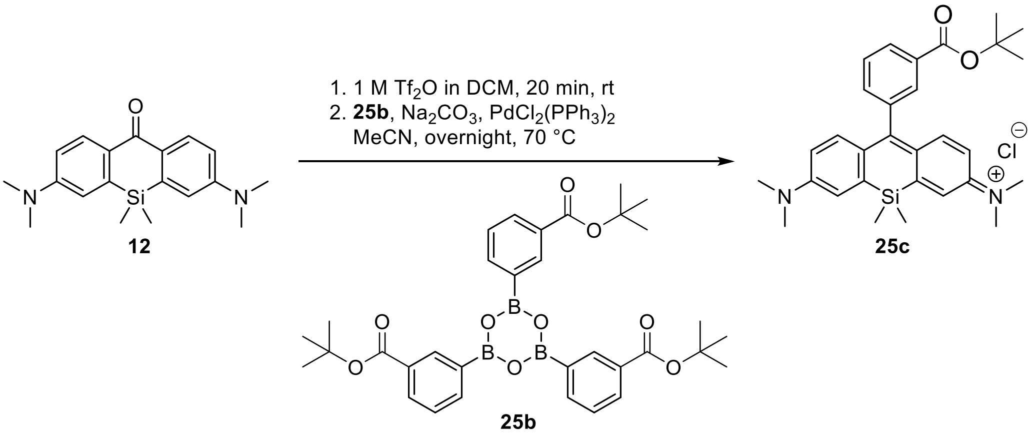

The synthesis was conducted as described in the general procedure 2.5. Xanthone **12** (50 mg, 154 µmol, 1.0 equiv) was treated with trifluoromethanesulfonic anhydride (170 µL, 170 µmol, 1 M in DCM, 1.1 equiv) in DCM (1 mL). Boroxine **25b** (94 mg, 154 µmol, 1 equiv) was used as boron source and bis(triphenylphosphine)palladium dichloride (12 mg, 154 µmol, 0.1 equiv), sodium carbonate (49 mg, 462 µmol, 3.0 equiv) were added and suspended in anhydrous acetonitrile (2 mL). Si-rhodamine **25c** was obtained as a blue solid in 43%[a] yield (50 mg, 95.5 µmol, 62%[a] brsm) after purification *via* column chromatography (silica gel, dichloromethane/methanol 99:1 to 90:10).

Using the same procedure 2.5 and [1,1′-bis(diphenylphosphino)ferrocene]dichloropalladium(II) (11.2 mg, 15.4 µmol, 0.1 equiv) as a catalyst provided rhodamine **25c** in 53% yield (53.0 mg, 102 µmol, 66% brsm).
[a]corrected yield, impurification with $[PPh_3(Ph\text{-}COO^{tert}Bu)]^+$

^{1}H NMR (600 MHz, CD_3OD, 300 K):
δ (ppm) = 7.51–7.50 (m, 2 H, H_{arom}), 7.36 (d, *J* = 2.8 Hz, 2 H, H_{arom}), 7.21 (s, 1 H, H_{arom}), 7.18–7.15 (m, 3 H, H_{arom}), 6.78–6.75 (m, 2 H, H_{arom}), 3.34 (s, 12 H, N-CH_3), 0.94 (s, 9 H, $C(CH_3)_3$), 0.60 (s, 6 H, Si-CH_3).

^{13}C NMR (151 MHz, CD_3OD, 300 K):
δ (ppm) = 181.3, 162.3, 155.7, 152.0, 151.7, 143.3, 139.7, 136.5, 129.7, 129.3, 128.9, 128.2, 122.1, 114.8, 40.9, 36.7, 26.6, −1.1.

R_f value (dichloromethane/methanol: 9:1) = 0.37

$C_{30}H_{37}N_2O_2Si^+$	Calculated m/z	found m/z
$[M]^+$	485.2619	485.2620

2.5.5 Synthesis of Si-rhodamine 30c

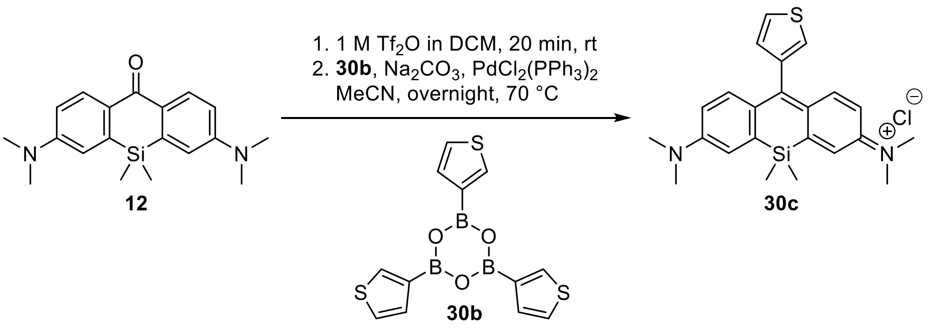

The synthesis was conducted as described in the general procedure 2.5. Xanthone **12** (30 mg, 92.5 µmol, 1 equiv) was treated with trifluoromethanesulfonic anhydride (102 µL, 102 µmol, 1 M in DCM, 1.1 equiv) in DCM (2 mL). Thienylboroxine **30b** (31 mg, 92.5 µmol, 1 equiv) was used as boron source and bis(triphenylphosphine)palladium dichloride (6.5 mg, 9.25 µmol, 0.1 equiv), sodium carbonate (29.4 mg, 278 µmol, 3 equiv) were added and suspended in anhydrous acetonitrile (2 mL). Si-rhodamine **30c** was obtained as a blue solid in 37%[a] yield (20 mg, 51.6 µmol, 56%[a] brsm) after purification via column chromatography (silica gel, chloroform/methanol 99:1 to 88:12).

Using the same procedure 2.5 and [1,1′-bis(diphenylphosphino)ferrocene]dichloropalladium(II) (6.80 mg, 9.25 µmol, 0.1 equiv) as a catalyst afforded rhodamine **30c** in 91% yield (35.9 mg, 84.2 µmol).
[a]corrected yield, impurification with $[PPh_3(3\text{-thienyl})]^+$

^{1}H NMR (400 MHz, CD_3OD, 400 K):
δ (ppm) = 7.70 (dd, *J* = 4.9 Hz, 3.0 Hz, 1 H, H_{arom}), 6.46 (dd, *J* = 3.0 Hz, 1.3 Hz, 1 H, H_{arom}), 7.34 (d, *J* = 2.9 Hz, 2 H, H_{arom}), 7.30 (d, *J* = 9.7 Hz, 2 H, H_{arom}), 7.13 (dd, *J* = 4.9 Hz, 1.3 Hz, 1 H, H_{arom}), 6.81 (dd, *J* = 9.7 Hz, 2.9 Hz, 2 H, H_{arom}), 3.35 (s, 12 H, N-CH_3), 0.59 (s, 6 H, Si-CH_3).
^{13}C NMR (400 MHz, CD_3OD, 300 K):
δ (ppm) = 155.7, 149.6, 143.3, 131.8, 130.4, 129.8, 129.3, 129.0, 122.1, 116.4, 114.9, 40.9, −1.1.

$\lambda_{max, Abs}$ = 653 nm;

$\lambda_{max, Em}$ = 672 nm;

Quantum yield: QY = 0.05.

R_f value (dichloromethane/methanol: 9:1) = 0.47

**$C_{23}H_{27}N_2SSi^+$**	Calculated m/z	found m/z
$[M]^+$	391.1659	391.1664

3. NMR-Spectra

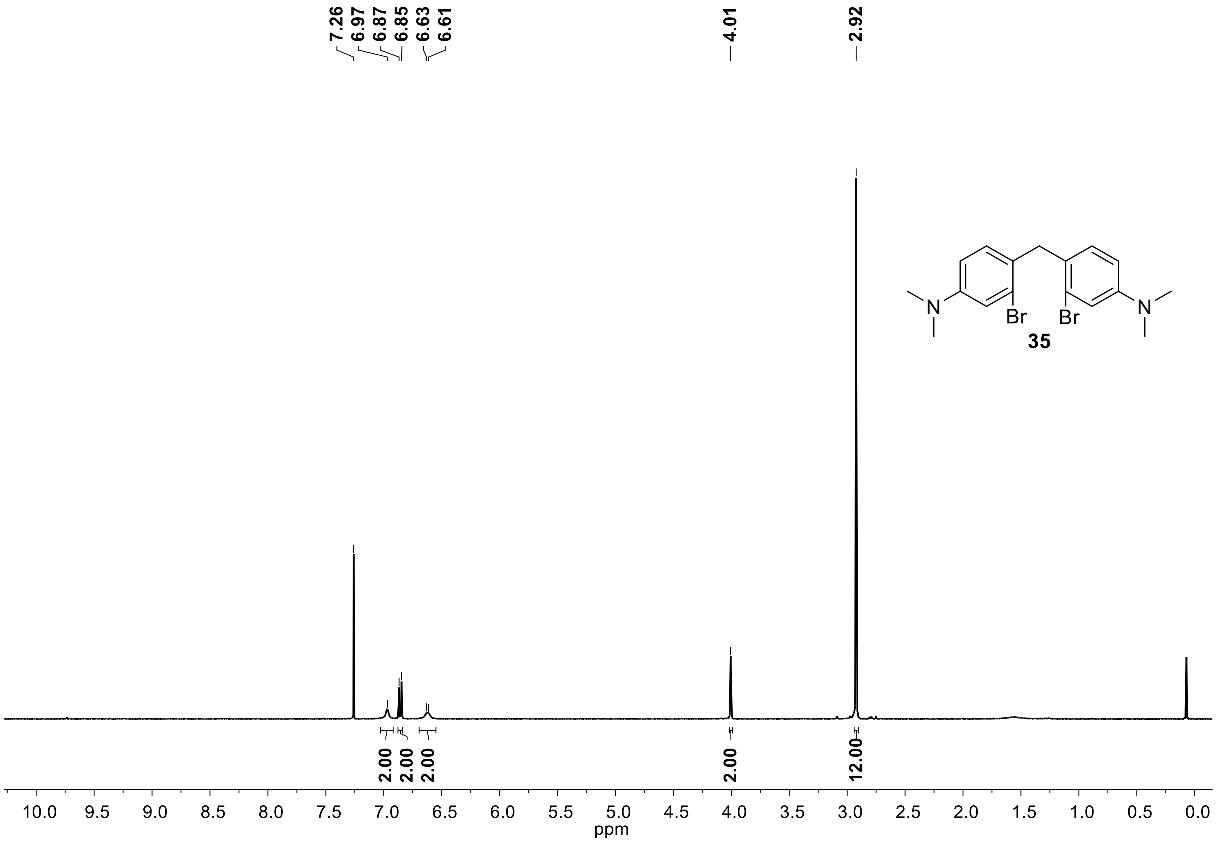

Figure S1: ^{1}H NMR-spectrum of **35** in chloroform-*d* (400 MHz, 300 K).

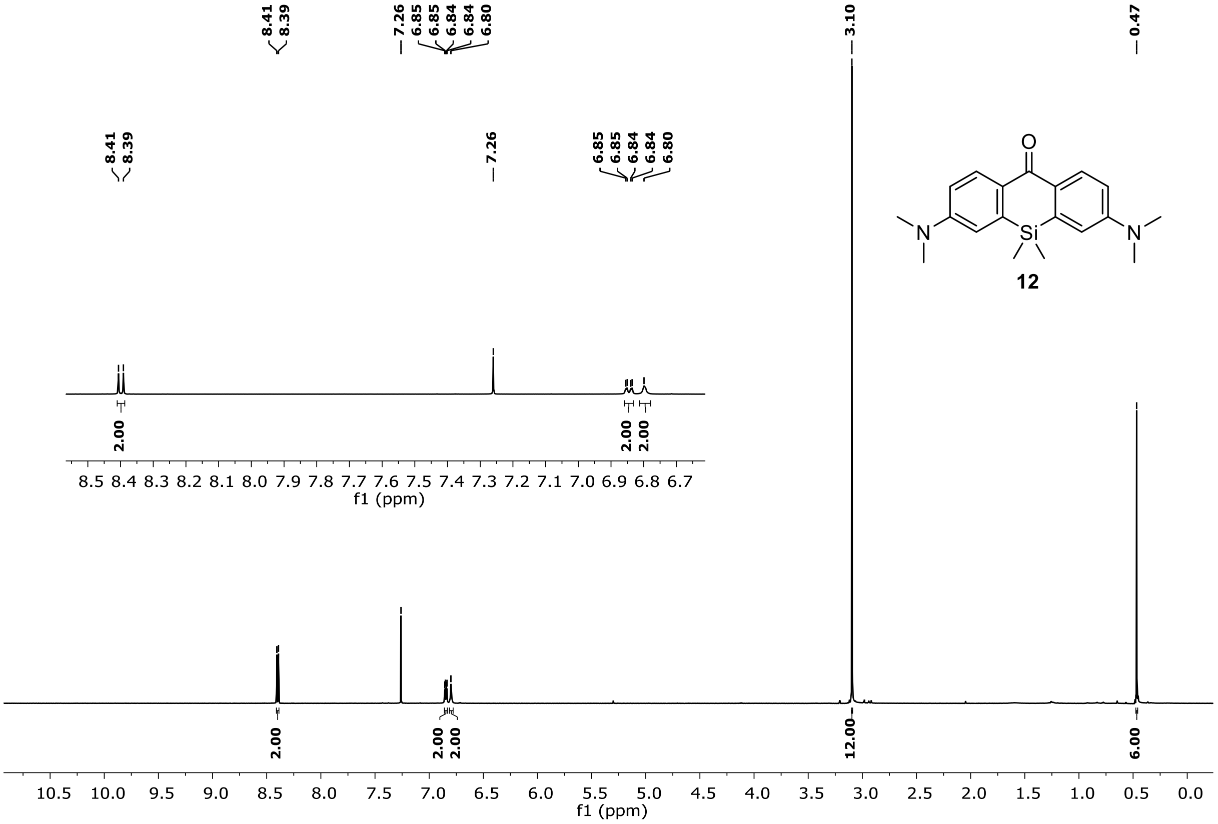

Figure S2: ^{1}H NMR-spectrum of **12** in chloroform-*d* (400 MHz, 300 K).

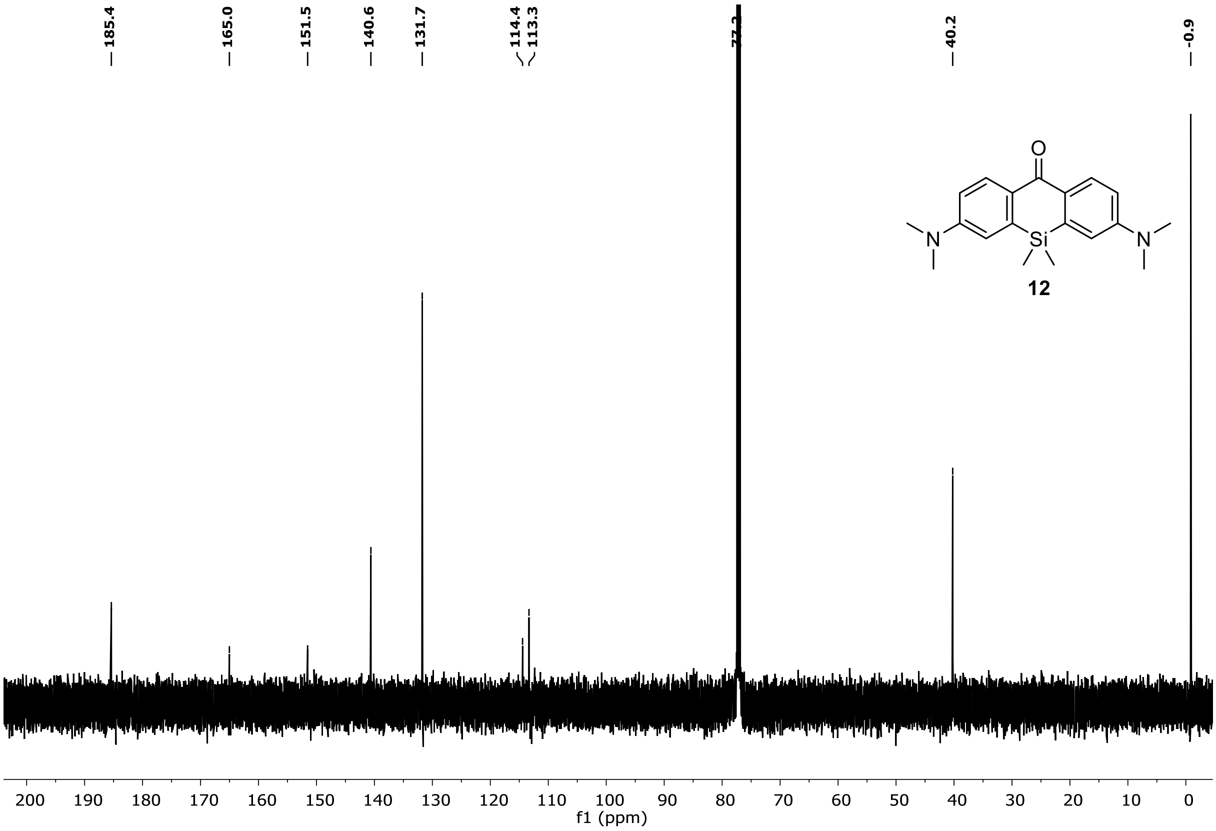

Figure S3: ^{13}**C{**^{1}**H} NMR-spectrum** of **12** in chloroform-*d* (151 MHz, 300 K).

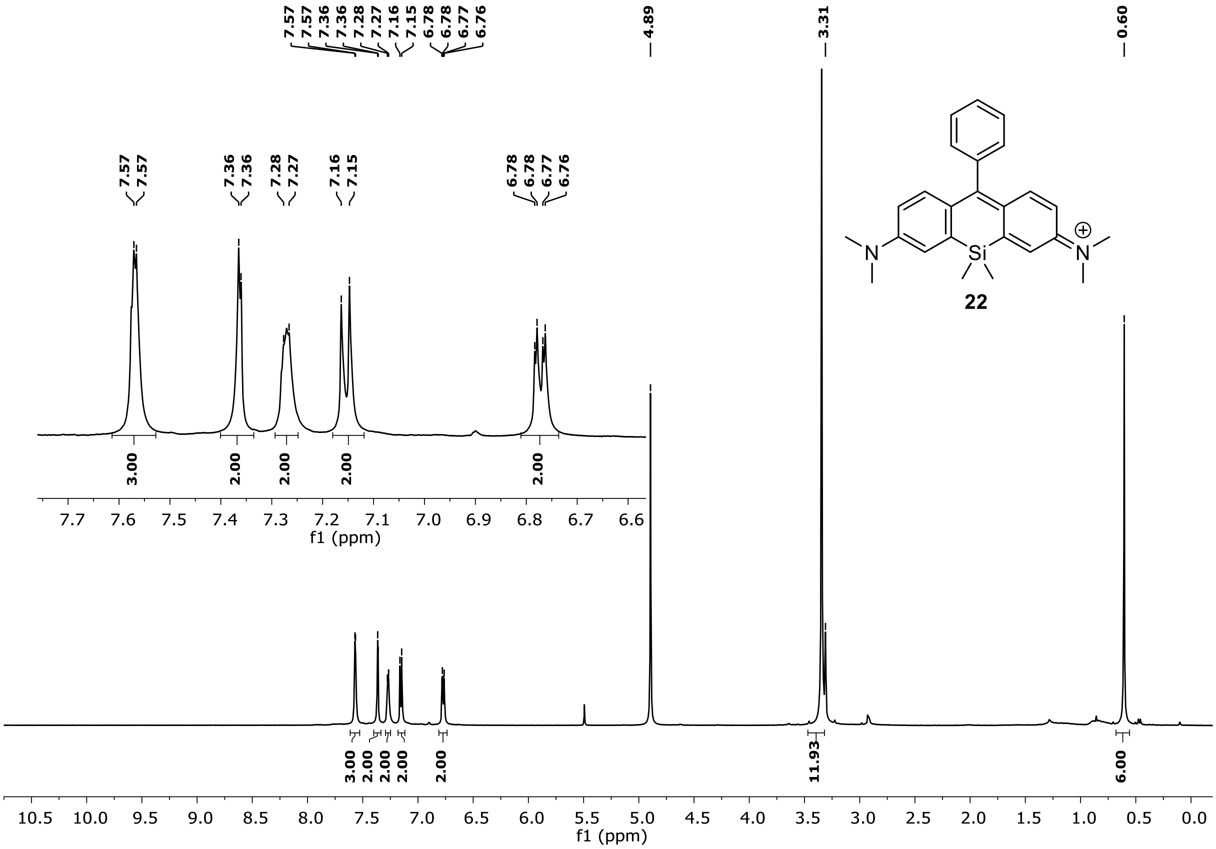

Figure S4: ^{1}H NMR-spectrum of **22** in methanol-d_4 (600 MHz, 300 K).

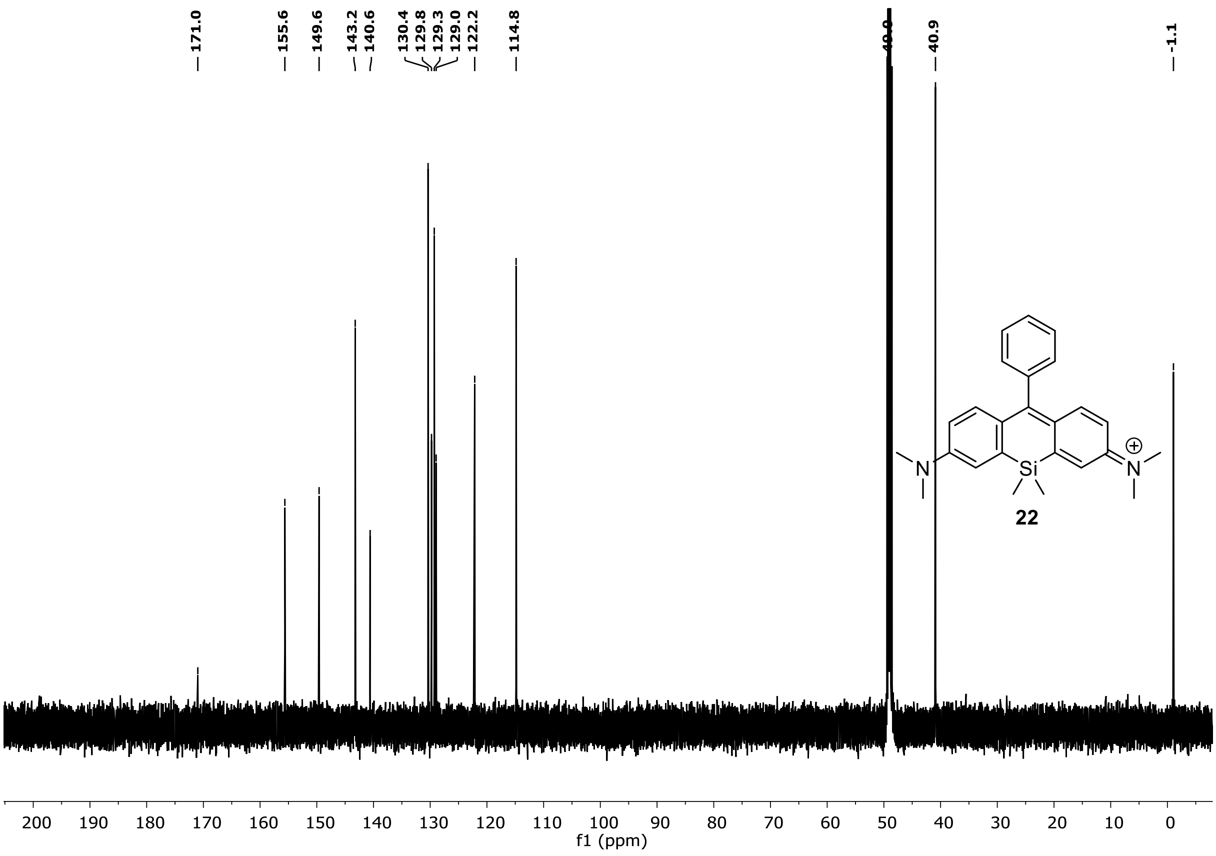

Figure S5: **^{13}C{^{1}H}-NMR-spectrum** of **22** in methanol-d_4 (151 MHz, 300 K).

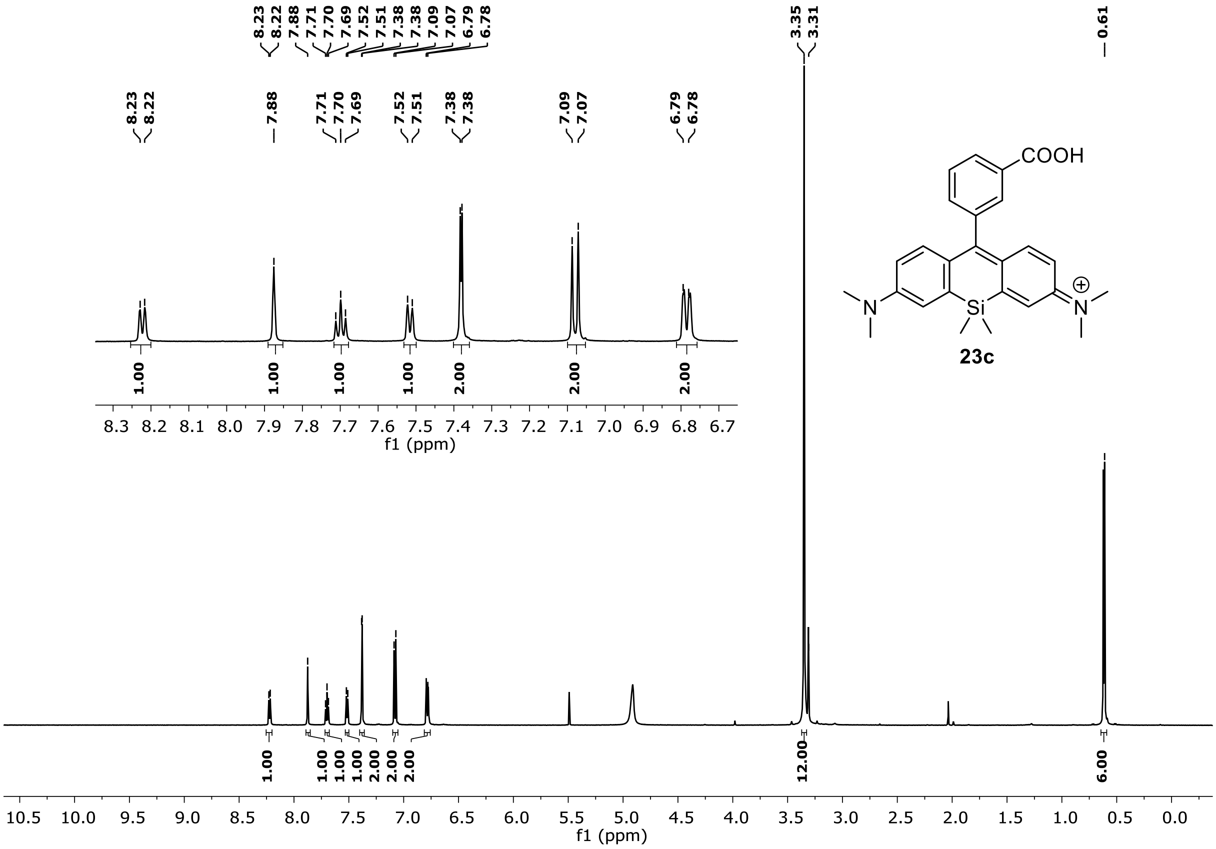

Figure S6: ^{1}H NMR-spectrum of **23c** in methanol-d_4 (600 MHz, 300 K).

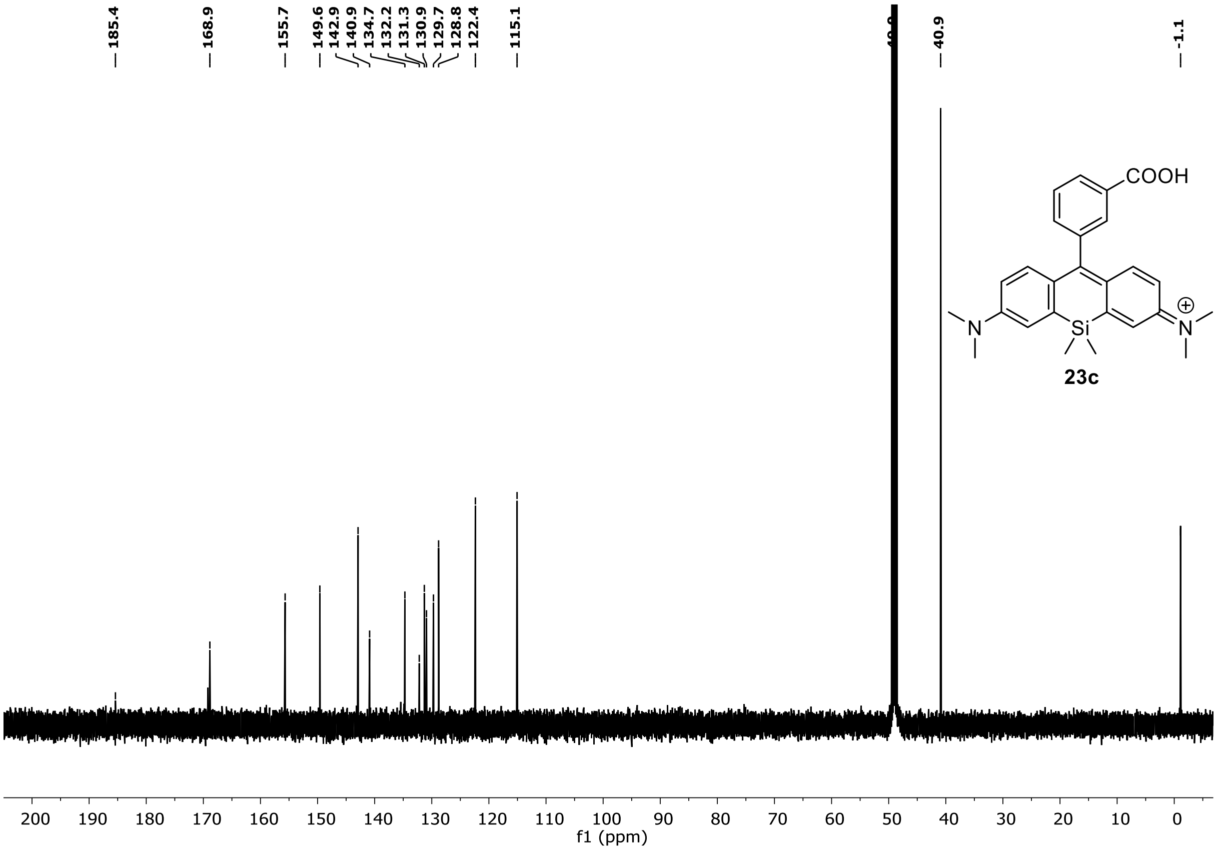

Figure S7: ^{13}**C{**^{1}**H} NMR-spectrum** of **23c** in methanol-*d*$_4$ (151 MHz, 300 K).

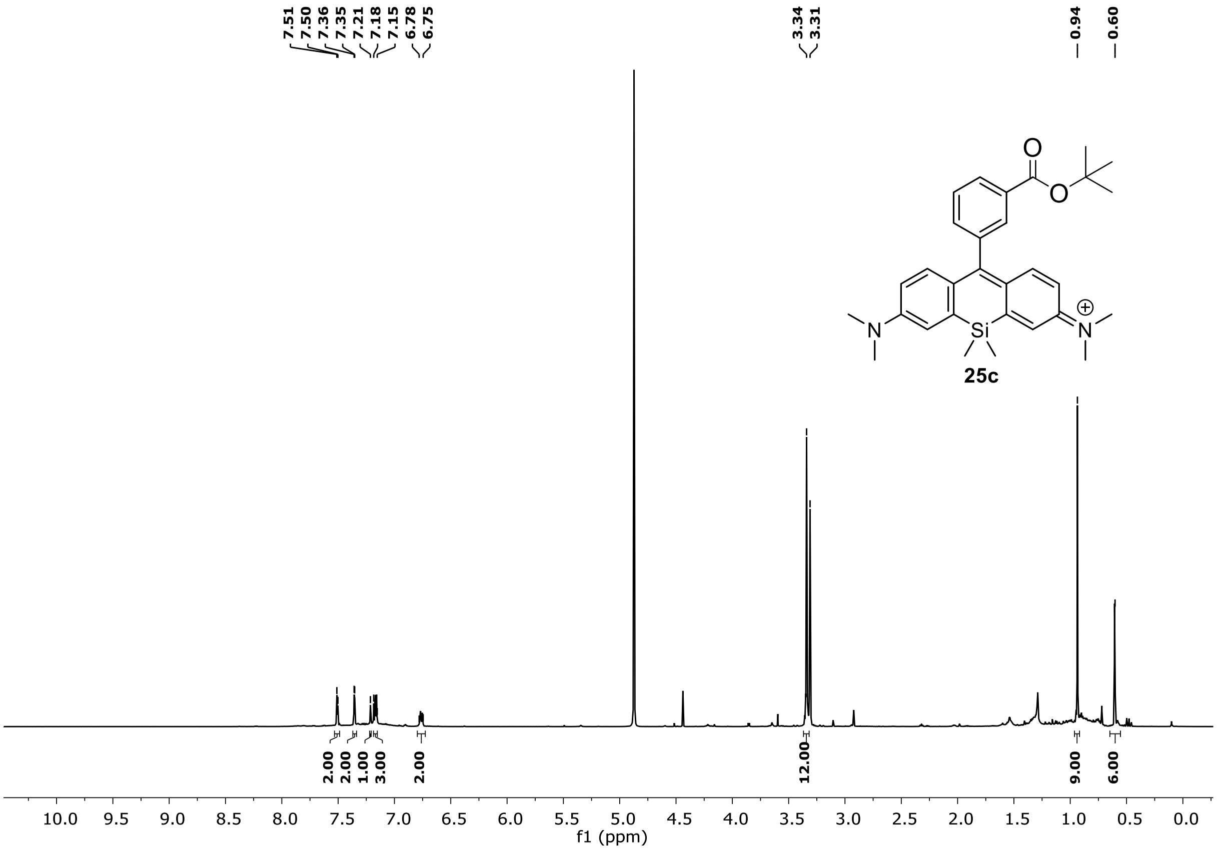

Figure S8: ^{1}H NMR-spectrum of **25c** in methanol-*d*$_4$ (400 MHz, 300 K).

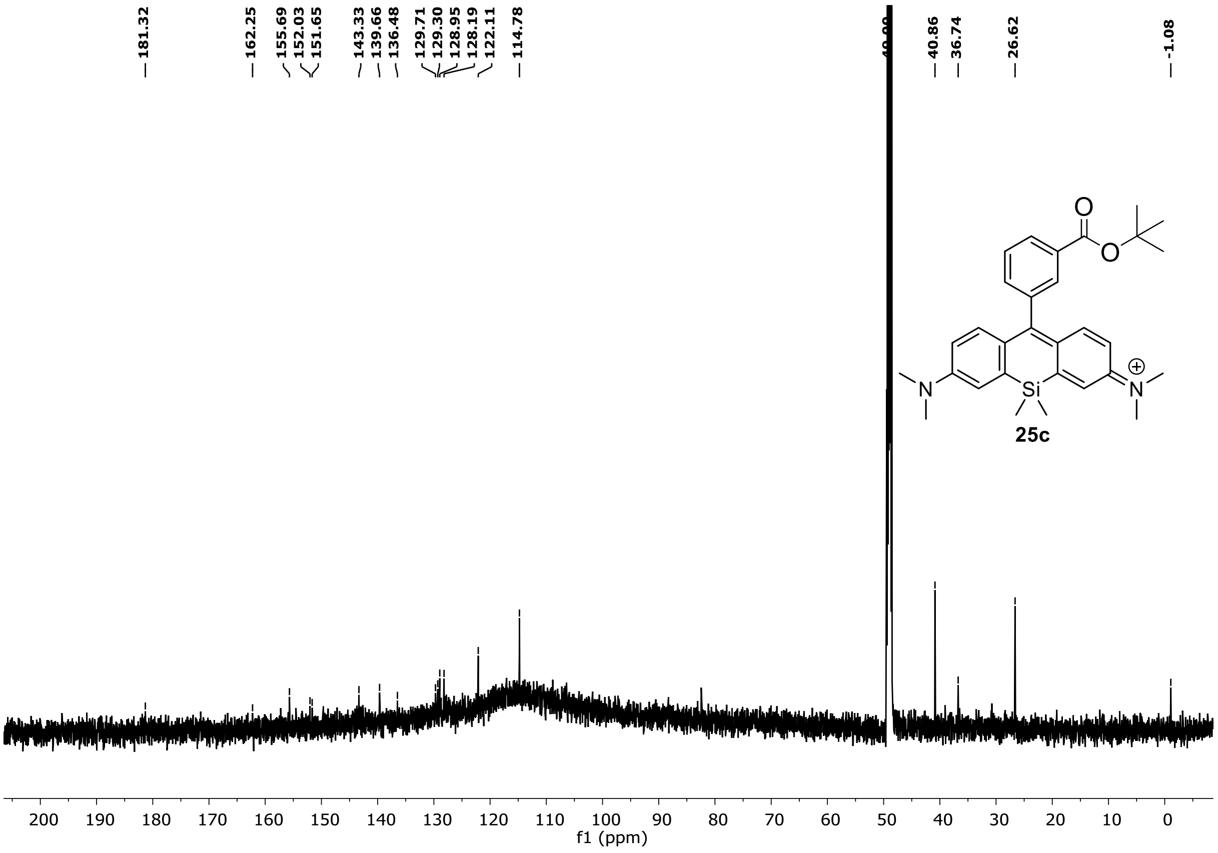

Figure S9: ^{13}**C{**^{1}**H} NMR-spectrum** of **25c** in methanol-*d*$_4$ (151 MHz, 300 K).

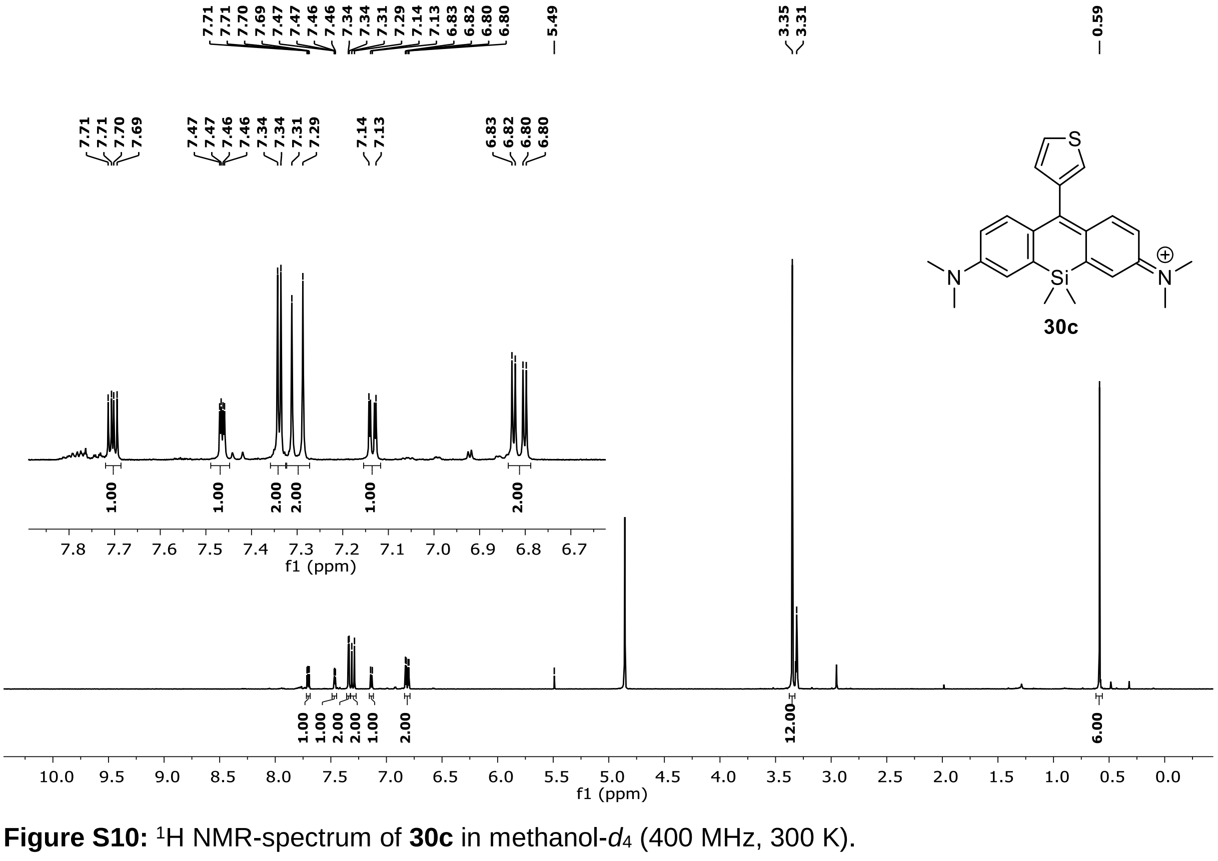

Figure S10: ^{1}H NMR-spectrum of **30c** in methanol-*d*$_4$ (400 MHz, 300 K).

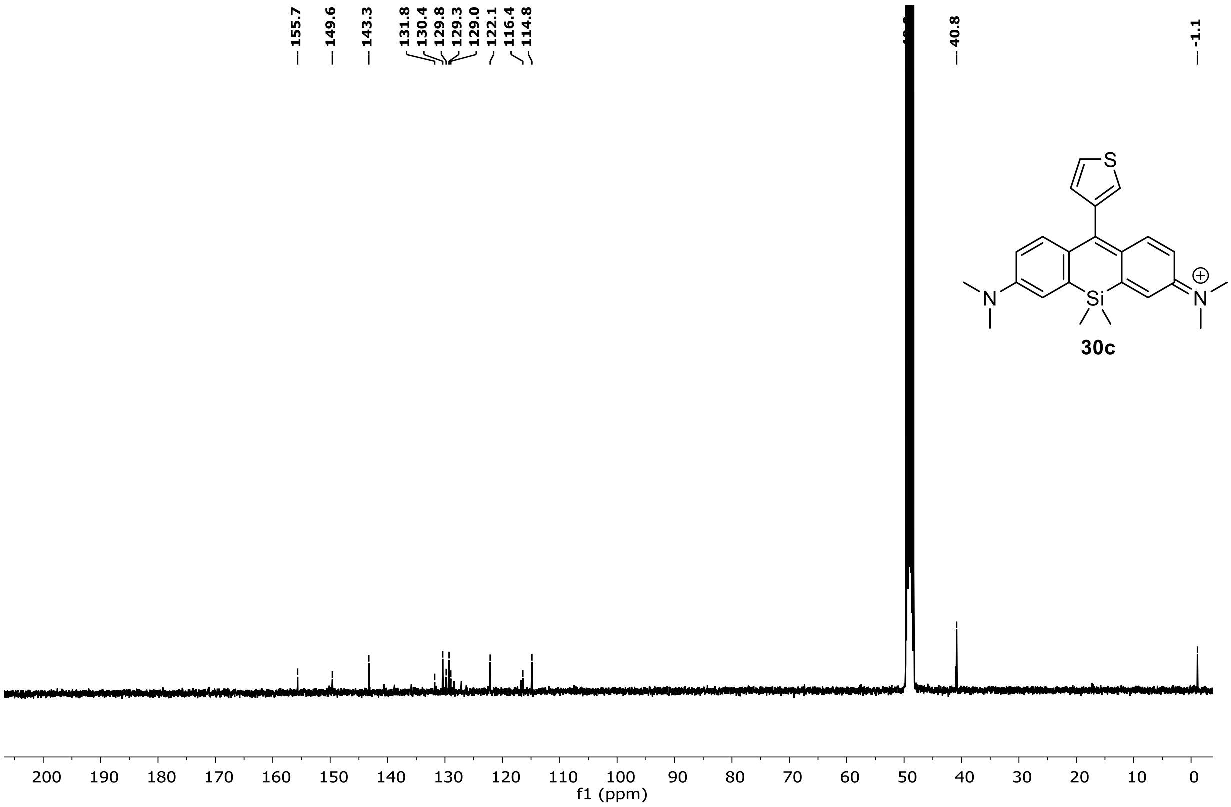

Figure S11: $^{13}C\{^{1}H\}$ **NMR-spectrum** of **30c** in methanol-d_4 (151 MHz, 300 K).

4. UV–vis/NIR-spectrum of compound 30c

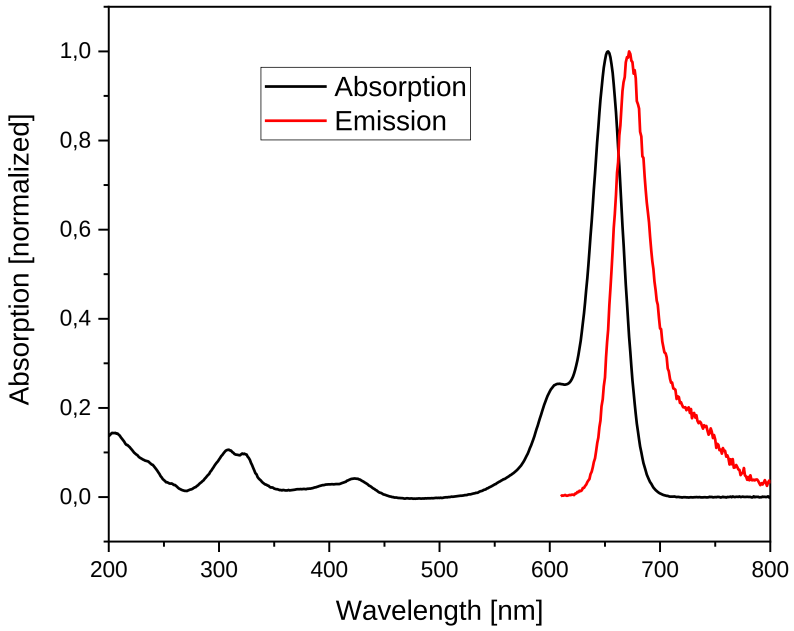

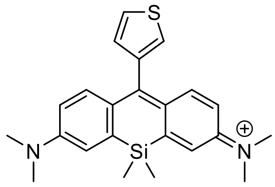

30c

$\lambda_{max,\ Abs}$ = 653 nm;

$\lambda_{max,\ Em}$ = 672 nm;

Quantum yield: QY = 0.05.

Figure S12: UV–vis/NIR-spectrum of **30c** in MeCN.
